# Prospective Study of Non-Autoimmune Insulin-Deficient Diabetes Subtype in Young Individuals in sub-Saharan Africa (PANDA study): study protocol

**DOI:** 10.1136/bmjopen-2026-127640

**Published:** 2026-09-25

**Authors:** Jean Claude Katte, Emmanuel Gwan, Mesmin Dehayem, Moffat Nyirenda, Andrew Hattersley, Angus Jones, Eugene Sobngwi, The PANDA Study Collaborators, Dana Dabelea

**Affiliations:** 1 Department of Clinical and Biomedical Sciences, University of Exeter Faculty of Health and Life Sciences, Exeter, UK; 2 Department of Non-Communicable Diseases Research, RSD Institute, Yaoundé, Cameroon; 3 National Obesity Centre and The Endocrinology and Metabolic Diseases Unit, Central Hospital of Yaounde, Yaoundé, Cameroon; 4 Department of Internal Medicine and Specialities, University of Yaounde I Faculty of Medicine and Biomedical Sciences, Yaoundé, Cameroon; 5 NCD Epidemiology, London School of Hygiene & Tropical Medicine, London, UK; 6 NCD Theme, MRC/UVRI and LSHTM Uganda Research Unit, Entebbe, Uganda; 7 Department of Clinical and Biomedical Sciences, University of Exeter Medical School, Exeter, UK; 8 Macleod Diabetes Centre, Royal Devon University Healthcare NHS Foundation Trust, Exeter, UK; 9 University of Exeter, Exeter, UK

**Keywords:** Adolescent, Africa South of the Sahara, Diabetes Mellitus, Type 1, Diabetes & endocrinology

## Abstract

**Abstract:**

**Introduction:**

Diabetes affects over 24 million people in Africa, with type 1 and type 2 diabetes being the most recognised forms. Recent studies in sub-Saharan Africa have identified a substantial proportion of children and young adults with clinically diagnosed type 1 diabetes (T1D) who lack evidence of islet autoimmunity despite severe insulin deficiency, suggesting a distinct non-autoimmune insulin-deficient diabetes subtype (NAID). This study aims to characterise the clinical phenotype, assess the underlying pathophysiology and longitudinal progression of this diabetes subtype.

**Methods and analysis:**

This is a prospective observational study comprising a cross-sectional comparative analysis and a 3-year longitudinal follow-up cohort. The study will recruit 355 children and young adults aged below 25 years with recently (diabetes duration ≤12 months) diagnosed T1D, with an anticipated estimate of 240 NAID and 115 autoimmune T1D cases. In addition, a minimum of 240 age-matched and sex-matched individuals without diabetes will be recruited as healthy controls. Participants will undergo detailed clinical phenotyping, laboratory assessment including islet autoantibody and C-peptide testing and biospecimen collection for biomarker analyses. A nested mechanistic substudy will include a mixed meal tolerance test and pancreatic MRI to assess pancreatic function and structure. Participants with diabetes will be followed for 3 years to assess longitudinal changes in endogenous insulin secretion, glycaemic response and acute metabolic complications. The study will provide the first prospective characterisation of NAID in sub-Saharan Africa and is expected to improve understanding of its clinical phenotype, natural history and underlying biology, thereby informing future diagnostic and therapeutic strategies.

**Ethics and dissemination:**

This study protocol was approved by the National Ethics Committee for Human Health Research (IRB No 2026/05/1930/CE/CNERSH/SP/SPA). All participants will provide written informed consent or assent with parental consent, where appropriate, before enrolment into the study. Research findings will be disseminated through peer-reviewed publications, policy briefs, stakeholder meetings and presentations at national and international scientific conferences.

**Trial registration number:**

ISRCTN15312527.

**STRENGTHS AND LIMITATIONS OF THIS STUDY**

- This prospective cohort study uses standardised longitudinal follow-up to assess clinical and biological changes over time.
- The comprehensive clinical, biochemical and imaging assessments provide multidimensional characterisation of participants using harmonised study procedures.
- The inclusion of age-matched and sex-matched healthy controls provides a robust comparator group for phenotypic analyses.
- The use of advanced MRI and mixed meal tolerance testing strengthens the measurement of pancreatic morphology and function.
- Recruitment from a single country and potential loss to follow-up may limit the generalisability and completeness of longitudinal analyses.

## Introduction

 Type 1 diabetes (T1D) is a chronic disease characterised by autoimmune-mediated destruction of pancreatic beta-cells resulting in absolute insulin deficiency. T1D is the most common form of diabetes in children and adolescents although it can also affect adults and the elderly.^1^ T1D accounts for about 5%–10% of all diabetes cases and is currently estimated to affect over 9 million individuals worldwide with the highest prevalence observed in Europe, North America and parts of the Caribbean.^2^ The incidence of T1D has increased globally over recent decades, although trends vary substantially. This variation is often attributed to the extent of the interaction between genetic susceptibility and environmental factors.^3^ Despite advances in understanding genetic susceptibility, the environmental and biological mechanisms triggering disease onset remain incompletely understood.^4^

Accurate classification of diabetes at diagnosis is increasingly challenging in children and young adults. For example, both T1D and type 2 diabetes (T2D) are heterogeneous with varied overlapping clinical presentation and disease progression patterns.^5^ T2D is a multifactorial condition characterised by hyperglycaemia resulting from insulin resistance and relative beta-cell deficiency. Making the correct diagnosis of T1D is critical as the treatment fundamentally differs from the treatment of T2D.^6^ Differentiating between these two diabetes types can be even more challenging in low-resource settings where diagnostic biomarkers such as islet autoantibody and C-peptide testing are often unavailable.^7 8^ This poses a huge challenge, where many people treated as having T1D may have other diabetes forms, given there is evidence of greater heterogeneity in African-ancestry populations.^8 9^

For decades, young people with insulin-requiring diabetes in Africa were assumed to have classical autoimmune T1D. However, recent work from us and others has challenged this assumption.^10 11^ For example, findings from the young-onset diabetes in sub-Saharan Africa (YODA) study conducted in Cameroon, Uganda, South Africa found that over 60% of children and young adults with a presumed diagnosis of T1D, where insulin-deficient, yet lacked the classical autoimmune and genetic features of T1D. This non-autoimmune insulin-deficient diabetes subtype (NAID) did not exhibit overt clinical and genetic features of T2D, suggesting that an alternative aetiology.^10^ Also, the YODA study was limited by its cross-sectional design and enrolled children and young adults with varying diabetes duration, meaning the true distinctive clinical features of NAID at diagnosis were not well elucidated. Therefore, robust longitudinal studies investigating the phenotype of this diabetes subtype at diagnosis and its progression will be paramount to providing evidence of its unique manifestation.

The prospective study of NAID subtype in young individuals in sub-Saharan Africa (PANDA) study is a longitudinal cohort designed to characterise this atypical diabetes form at diagnosis. By integrating detailed clinical phenotyping, longitudinal metabolic follow-up, pancreatic imaging and biospecimen collection, the PANDA study aims to provide the first comprehensive prospective characterisation of non-autoimmune diabetes forms in a young African population. The findings are expected to improve clinical recognition, inform optimal management and establish a platform for future mechanistic studies into this emerging diabetes subtype.

## Methods and analysis

### Study design

This is a prospective observational study comprising two components: a cross-sectional comparative component (including a nested substudy) and a longitudinal cohort analysis with a 3-year follow-up. The cross-sectional component will characterise the clinical, metabolic and pathophysiological mechanisms of NAID at diagnosis and compare these with autoimmune T1D and healthy controls. The longitudinal component will assess disease progression, including trajectories of beta-cell function (beta-cell decline), glycaemic control, insulin requirements and rates of acute metabolic complications (severe hypoglycaemia and diabetic ketoacidosis (DKA)) among participants with diabetes only.

### Study setting

The study will be conducted in Cameroon and will leverage the national T1D management programme to facilitate participant recruitment and follow-up. The national T1D management programme provides comprehensive care including free insulin treatment for all children and young adults diagnosed with diabetes before 25 years, supported through Changing Diabetes in Children (CDiC) initiative.^12^ Recruitment will take place through the network of 11 specialised T1D clinics which has been set up as part of CDiC, distributed across the 10 regions of the country. The nationwide network of specialised clinics provides an established infrastructure for the identification, recruitment and long-term follow-up of children and young adults with recently diagnosed diabetes, making it well suited for a prospective cohort study. The specialised T1D clinic at the Yaoundé Central Hospital will serve as the main coordinating centre for study activities.

### Study population

The study population will include children and young adults with a clinician diagnosis of T1D before 25 years of age and age-matched and sex-matched individuals without diabetes recruited as healthy controls. Participants with diabetes will be identified either from existing registry data at the specialised clinic, referral from other healthcare facilities or first-time outpatient consultations. A recent registry data examination (3 August 2026) revealed that 166 individuals had a clinician diagnosis of T1D across the 11 specialised clinics since January 2025. Of these, 42 have been assessed eligible for direct recruitment into the study. Healthy controls will be unrelated to participants with diabetes and will be recruited from the same communities to provide a representative comparator population for cross-sectional analyses.

### Eligibility criteria

Participants included in the study must meet the following criteria:

- Clinician diagnosis of T1D before 25 years of age.
- Diabetes duration of less than 12 months at enrolment into the study.
- Currently treated on insulin alone.
- Ability and willingness to provide written informed consent. For participants below the age of legal consent, written informed assent will be obtained where appropriate, alongside written informed consent from a parent or legal guardian.

Participants will be excluded from the study if they:

- Are critically ill and require urgent hospital admission or emergency medical intervention.
- Are pregnant at the time of enrolment.

### Sampling strategies

Research team investigators (EG and LKJ) will review the registry records of patients diagnosed with T1D within the last year against study eligibility criteria and invite individuals who match our inclusion criteria. This will provide a pool of participants that will immediately be recruited into the study. Subsequently, research team investigators will work closely with nurses at the specialised clinics to recruit referred or new cases over the course of the study period. The 3-year follow-up longitudinal cohort component of the study will leverage the specialised clinic review calendar of the participants, which is usually every 3 or 6 months to plan for subsequent visits.

### Data collection

Data will be collected by trained research assistants using prestructured and pretested data collection forms, which will capture key sociodemographic data (age, sex, level of education, household assets, personal income, household income and occupation), early-life data (place of birth and childhood malnutrition), lifestyle (physical activity and diet patterns), family history of diabetes (siblings and parents), medical history of other chronic illnesses, history of diabetes (age at diabetes diagnosis and circumstances of diabetes diagnosis), history of medication use and vaccinations. Anthropometric measurements (weight and height, with calculation of body mass index (BMI), waist and hip circumferences, mid-upper arm circumference and skin fold) and bioimpedance parameters (total body fat, fat mass and free fat mass) will be measured using standardised and validated instruments and recorded into the appropriate sections in the data collection forms.

### Study procedures

Eligible participants will be invited for a formal study visit (baseline visit or visit 1) and the subsequent (follow-up) visits will be planned well in advance at a time agreed between the research team investigators, the participants and their clinicians. A subsample of those participating in the baseline visit will be invited to a substudy at a separate date. Figure 1 presents a summary of the participants’ flow and study visits.

**Figure 1:**
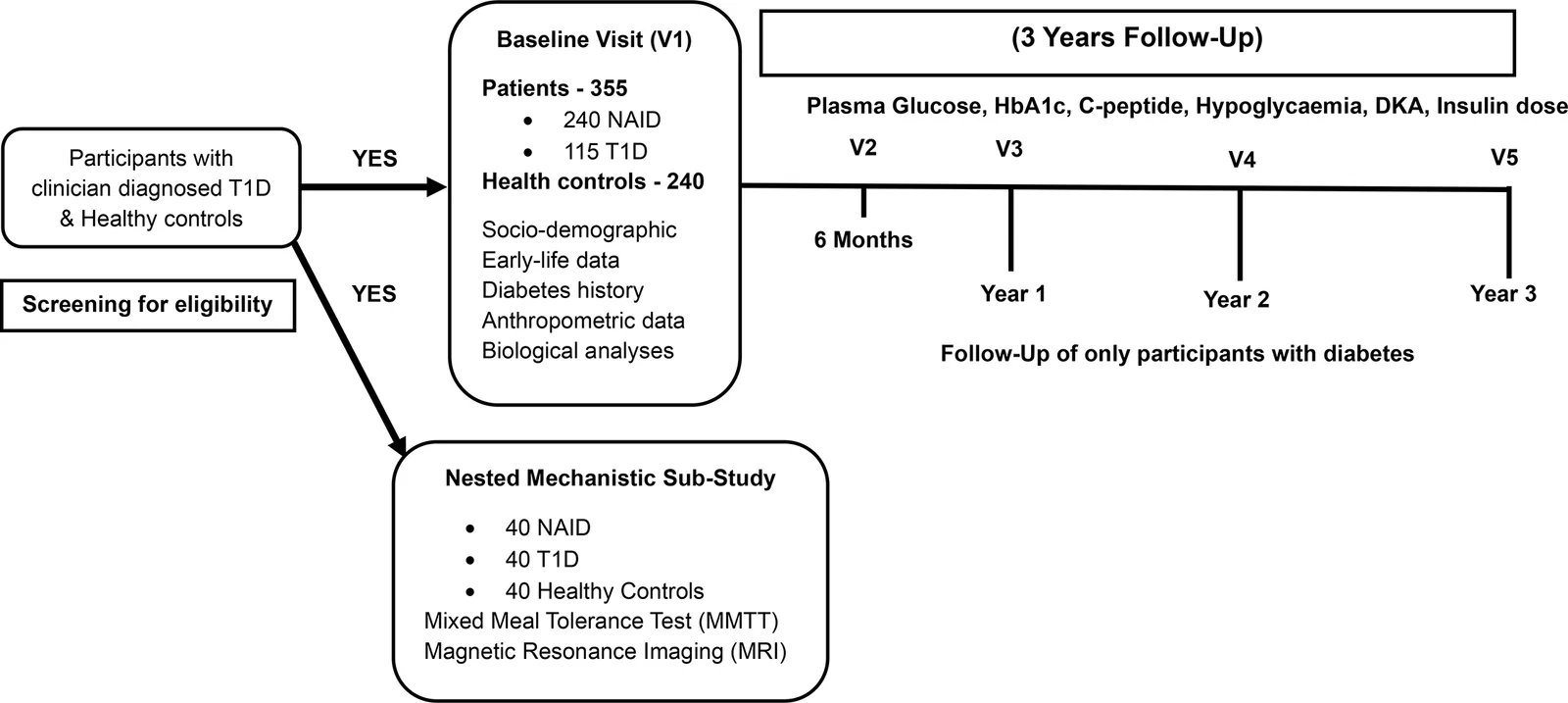
Schematic representation of the participants’ flow and the timelines of the different study visits. DKA, diabetic ketoacidosis; HbA1c, glycated haemoglobin; NAID, non-autoimmune insulin-deficient diabetes; T1D, type 1 diabetes.

### Invitation

Invitations will be made via telephone following examination of the clinic registry to all eligible participants. Clinician-diagnosed referred and newly recognised cases at outpatient consultations will also be invited to enrol in the study. They will be provided with the necessary information regarding the study. They will be encouraged to ask questions if they do not understand any information regarding the study. Those who accept to participate in the study will be required to provide a written consent form (or assent where necessary) and given an appointment for the baseline study visit. They will be informed that they can choose to discontinue their participation in the study at any time without their decision affecting their routine care. Participants will be encouraged to eat and administer their usual insulin treatment as prescribed by their doctor or nurse at home before attending the appointment.

### Baseline visit (visit 1)

On the day of the appointment, research team investigators will verify the participants’ identity, eligibility criteria and make a note of the time of last meal. Visit 1 will consist of 3 phases: in-depth interview, clinical examination and biospecimen collection, which will last an estimated duration of 45 min.

Phase 1 will consist of an in-depth interview and review of participant medical records. The interview will be conducted using a prestructured data collection form administered by one of the research assistants.

Phase 2 will consist of a thorough clinical examination by the investigator and the measurement of anthropometric and bioimpedance parameters.

Phase 3 will consist of the sampling of biospecimens (saliva, blood, urine and stool). Saliva samples will be collected in a saliva collection tube. Whole (venous) blood samples will be collected within 5 hours of a carbohydrate-containing meal. Where attending participants have fasted >5 hours, a carbohydrate-containing snack will be offered, and blood sampling delayed until 1 hour after snack consumption. A urine sample will be collected in a sterile urine collection pot. Participants who are unable to provide an immediate stool sample on the day of examination will take the sterile pot home and bring a stool sample on any other available day or the day of their next clinic visit.

### Longitudinal follow-up visits (visit 2, visit 3, visit 4 and visit 5)

Research team investigators will plan these visits leveraging the routine follow-up calendar of the patients in the specialised clinics to minimise discordant clinic appointments. Visits 2, 3, 4 and 5 are planned for 6 months, 1 year, 2 years and 3 years after the baseline visit. On these visits, research team investigators will administer a short data collection form capturing data on clinical features, anthropometric characteristics, acute metabolic complications (severe hypoglycaemia and DKA). Finally, at each follow-up visit, blood samples will be collected for full blood count, glycated haemoglobin (HbA1c), plasma glucose and C-peptide determination. These visits are estimated to last approximately 30 min at any time.

### Anthropometric and bioimpedance measurements

Weight will be measured in the standing position to the nearest 0.1 kg using a calibrated electronic weighing device (TANITA. TANITA Corporation 1-14-2 Maeno-Cho, Tabashi-ku, Tokyo, Japan). Standing height will be measured using a standard wall stadiometer to the nearest 0.1 cm. BMI will be calculated using the Quetelet’s formula: weight/height^2^ and expressed in kg/m^2^ to the nearest 1 decimal place. Waist circumference will be measured using a normal measuring tape at the midpoint between the lower costal margin and the anterior superior iliac spine during expiration to the nearest 0.1 cm. The average of two measurements will be used for data entry. Hip circumference will be measured with the same measuring tape around the most protruding part of the buttocks. Mid-upper arm circumference will be measured using a standardised tape to the nearest 0.1 cm at the midpoint between the olecranon and tip of the acromion. TANITA instrument used for weight measurements will also be used to capture and record the key relevant bioimpedance parameters (total body fat, free fat mass).

### Biospecimen collection, processing, analysis and storage

Table 1 summarises the biospecimens to be collected, their intended analyses and storage conditions. Saliva will be collected using a standardised saliva collection tube (GeneFix Assisted DNA Saliva Collector; Isohelix, Kent, UK) for genomic DNA extraction and subsequent genetic analyses. Whole blood collected into sodium fluoride and EDTA tubes will be used for immediate measurement of plasma glucose, full blood count and HbA1c. Additional venous blood samples will be collected in EDTA and serum separator tubes (SST), processed immediately by centrifugation, aliquoted into plasma and serum cryovials, and stored at −80°C pending batch analysis of C-peptide, islet autoantibodies and other predefined biomarkers. Mid-stream urine samples will be collected in sterile urine containers for immediate dipstick testing, with aliquots stored at −80°C for future biomarker analyses. Stool samples will be collected using specialised collection tubes and stored at −80°C pending batch analyses.

**Table 1:** Table of type of biospecimen collected, analyses and storage for baseline samples

Biospecimen	Collection tube	Immediate analyses	Batch analyses	Storage
Whole blood	NaF/EDTA	Glucose, FBC, HbA1c	-	-
Plasma	EDTA	-	C-peptide and proinsulin	Storage at −80°C for future mechanistic analyses
Serum	SST	-	Islet autoantibodies (GADA, IA-2A, ZnT8A), creatinine, lipid panel, lipase, amylase, trypsinogen	Storage at −80°C for future mechanistic analyses
Saliva	GeneFix Saliva Collection tube	-	DNA extraction, array genotyping and calculation of T1D and T2D PRSs	Storage of DNA at −80°C for future genomic sequencing
Urine	Sterile pot	Dipstick	Microalbuminuria	Storage at −80°C for future metabolite analyses
Stool	Collection tube	-	Faecal elastase-1	Storage at −80°C for future mechanistic and metagenomics analyses

FBC, full blood count; HbA1c, glycated haemoglobin; NaF, sodium fluoride; PRS, polygenic risk score; SST, serum separator tube; T1D, type 1 diabetes; T2D, type 2 diabetes.

### Cross-sectional substudy

A nested mechanistic substudy will be undertaken to investigate pancreatic morphology, endocrine and exocrine function among participants NAID, autoimmune T1D and healthy controls. The substudy will recruit 120 participants comprising 40 participants with NAID, 40 participants with autoimmune T1D, and 40 age-, sex-matched and BMI-matched healthy controls. Eligibility will be restricted to participants receiving care at the Yaoundé Central Hospital because MRI facilities and clinical research unit required for overnight admission and metabolic testing are located at this site.

The MRI will be conducted using an MRI PHILIPS PRODIVA 1.5 TESLA equipment. The protocol for pancreatic MRI imaging will follow the protocol described by Virostko and colleagues in 2021 called the Multicentre Assessment of the Pancreas in T1D, MAP-T1D.^13^ MRI will be used to quantify pancreatic volume and volume index and assess pancreatic morphology, including pancreatic fat infiltration and fibrosis.

The mixed meal tolerance testing (MMTT) will be conducted using a weight-based liquid meal (160 mL of Fortisip Compact or equivalent) drunk within 5–10 min.^14^ The liquid meal drink contains: 100 mL/carbohydrate 29.7 g, protein 9.6 g and fats 9.3 g. Timed venous blood samples, 10 min prior to ingestion (t=0), and at 30, 60, 90 and 120 min will be collected in EDTA (placed on ice) and SST tubes, immediately centrifuged, separated, and stored at −80 °C pending batch metabolic investigations. Batch metabolic analysis will focus on the determination of predefined endocrine (C-peptide, proinsulin, glucagon) and exocrine (trypsinogen) biomarkers.

### Definitions

Clinician-diagnosed T1D will be defined by healthcare provider diagnosis of T1D in individuals developing diabetes below 25 years of age according to criteria for diagnosis of diabetes in non-pregnant individuals by the American Diabetes Association guidelines,^6^ with fasting plasma glucose ≥7.0 mmol/L or a random plasma glucose ≥11.1 mmol/L in individuals with classic symptoms of hyperglycaemia or hyperglycaemic crisis and on permanent insulin treatment. Autoimmune T1D will be confirmed in those with clinician-diagnosed T1D who are positive for at least one islet autoantibody (GADA, IA-2A, ZnT8A), irrespective of plasma C-peptide levels. NAID will be confirmed in participants with clinician-diagnosed T1D, negative for all islet autoantibodies (GADA, IA-2A, ZnT8A) and insulin-deficient or requiring (defined as plasma C-peptide levels ≤600 mmol/L).

### Study outcomes

The primary study outcomes are differences in key clinical characteristics; endogenous insulin secretion measured by C-peptide, HbA1c, BMI and age at diabetes diagnosis between participants with NAID and autoimmune T1D. The main study outcome measure that drove the sample size calculation (see below) was endogenous insulin secretion. Secondary study outcomes will be differences in risk factors contributing to the development of NAID focused on comparing clinical/biological and sociodemographic characteristics between participants with NAID and healthy controls. Rates of acute metabolic complications, including severe hypoglycaemia and DKA among participants with NAID compared with those with autoimmune T1D. Differences in pancreatic morphology and endocrine/exocrine pancreatic function between NAID and autoimmune T1D and NAID and healthy controls.

### Sample size determination

For the baseline descriptive and prospective analytical components of the study, we will recruit a total of 355 participants with recently (diabetes duration less than 12 months) clinician-diagnosed T1D in Cameroon. This sample size was calculated using data from our previous study in Cameroon, which demonstrated a ratio of 2.3:1 between participants with NAID and autoimmune T1D. Based on an SD of 0.40 for endogenous insulin secretion, a statistical power of 90% and two-sided significance level of 5%, a minimum sample of 324 participants was required. Allowing for a 10% loss to follow-up, the final target sample size of 355 participants, comprising an anticipated approximate number of 240 participants with NAID and 115 with autoimmune T1D. In addition, a minimum of 240 age-matched and sex-matched healthy controls will be recruited to provide comparative population data based on the anticipated number of participants with NAID, the principal study population of interest.

For the nested mechanistic substudy, we estimated a sample size of 40 participants per group informed by previous pancreatic imaging studies showing differences in pancreatic volume between healthy individuals and participants with T1D, corresponding to a standardised effect size of approximately 0.64 and assuming 80% power and a two-sided significance level of 5%. The substudy will therefore recruit 40 participants with NAID, 40 participants with autoimmune T1D, and 40 age, sex and BMI-matched healthy controls. All 120 participants enrolled in the substudy will undergo MMTT, and pancreatic MRI will be performed to investigate endocrine and exocrine pancreatic dysfunction and assess pancreatic morphology and architecture.

### Data management

The lead investigator and research study coordinator will directly oversee data management throughout the course of the study. Data will be collected and managed electronically using REDCap (Research Electronic Data Capture), which is a secure web-based application for building and managing databases.^15^ This will be hosted by one of the collaborating institutions (RSD Institute), based in Cameroon. A REDCap-based data management system will reduce data entry errors, enhance rapidity of data collection and processing in real-time. This will facilitate a monthly review of the collected data to monitor the progress of the study.

### Statistical analysis

Statistical analysis will be performed using STATA V.19SE (StataCorp) by the principal investigator. Continuous variables will be summarised as mean (SD) or median (IQR), according to distribution. Categorical variables will be presented as frequencies and percentages. Group comparisons will be achieved using Student’s t-test or Mann-Whitney U test, as appropriate. To identify the distinctive characteristics of NAID, we will compare the clinical and biological characteristics of participants with NAID to those with autoimmune T1D. To determine critical risk factors contributing to the development of NAID, we will compare exposure variables and characteristics (clinical and biological) of participants with NAID to the healthy controls. Multivariable regression models will be used to identify the distinctive characteristics and risk factors associated with NAID after adjusting for predefined confounders. Mixed-effect models will be used to assess longitudinal C-peptide trajectories (beta-cell decline) between those with NAID versus autoimmune T1D. In the subset of participants undergoing the MMTT and MRI assessments, pancreatic function and morphological parameters will be compared between participants with NAID versus autoimmune T1D and NAID versus healthy controls. A two-sided p<0.05 will be considered statistically significant for all analyses, except if a corrective statistical measure is used to adjust for multiple testing where necessary.

### Patient and public involvement

People living with T1D, patient advocates, healthcare professionals and representatives of the diabetes community in Cameroon were actively involved in the development of this study. Their input helped ensure the research questions, study procedures and implementation strategy reflected local priorities and were acceptable to the target population. Patient and public involvement will continue throughout the study. A diabetes community support leader with lived experience of T1D is a member of the research team and will facilitate participant engagement, community liaison and communication between the investigators and the wider diabetes community. Patient representatives will continue to provide advice during the study implementation and will contribute to the interpretation and dissemination of the findings.

### Study status

This manuscript describes protocol version 2.0 (March 2026). The study began in January 2026 with the writing of the protocol for ethical approval submission, development of standard operating procedures and training of research team members. Participant recruitment across the different components of the study is scheduled to commence in October 2026, with recruitment expected to continue until December 2030. Follow-up of enrolled participants will continue in accordance with the study protocol. At the time of manuscript submission, participant recruitment had not yet commenced.

## Discussion

This study aims to characterise a previously unrecognised atypical form of non-autoimmune diabetes (NAID) which has previously been shown to be common in children and young adults in sub-Saharan Africa. We currently do not know how this new subtype differs from autoimmune T1D clinically, especially at diagnosis and whether the progression of the disease would be different. We also do not know what the specific aetiologic drivers of this condition would be. This study will therefore for the first time characterise this new subtype of diabetes at diagnosis, investigate the progression of the condition and assess the contribution of environmental exposures to the development of this novel subtype of diabetes in children and young adults in Cameroon. This data is timely and will contribute to discoveries around the heterogeneity of diabetes globally.

### Ethics and dissemination

This study protocol was approved by the National Ethics Committee for Human Health Research (IRB No 2026/05/1930/CE/CNERSH/SP/SPA) and the Centre Regional Ethics Committee for Human Health Research (IRB No 0729/CRERSHC/2026). All participants will provide a written informed consent before enrolment into the study. As per local Cameroonian guidance, written informed assent will be obtained from minors (under 21 years); written informed consent from their legal representatives (parents or tutors), as well as directly from participants aged over 21 years. Research findings will be disseminated via workshops, seminars, knowledge transfer and exchange meetings, and through poster and oral presentations at local and international conferences, and as policy briefs and publications in peer-reviewed scientific journals.
