# Supplementary material for: Prospective Study of Non-Autoimmune Insulin-Deficient Diabetes Subtype in Young Individuals in sub-Saharan Africa (PANDA study): study protocol
Source: BMJ Open. 2026 Sep 25;16(9):e127640. doi: 10.1136/bmjopen-2026-127640 (PMC13630088; doi:10.1136/bmjopen-2026-127640)
Supplement: Supplementary file 1 [file bmjopen-2026-127640.reviewer_comments.pdf]

PEER REVIEW HISTORY

BMJ Open publishes all reviews undertaken for accepted manuscripts. Reviewers are asked to complete a checklist review form and are provided with free text boxes to elaborate on their assessment. These free text comments are reproduced below.

ARTICLE DETAILS

Title (Provisional)

Prospective Study of Non-Autoimmune Insulin-Deficient Diabetes Subtype in Young Individuals in sub-Saharan Africa (PANDA Study): Study Protocol

Authors

Katte, Jean Claude; Gwan , Emmanuel; Dehayem, Mesmin; Nyirenda, Moffat; Hattersley, Andrew; Jones, Angus; Sobngwi, Eugene; Collaborators, PANDA Study

---

This article was not externally reviewed at BMJ Open. Protocols that have been independently assessed prior to submission to BMJ Open are usually fast-tracked to publication on the grounds that further substantial changes will not be possible. This independent assessment will usually be external, independent review for both a substantial grant award from a non-commercial or government funder and ethics approval.
